# Rad59 regulates association of Rad52 with DNA double-strand breaks

**DOI:** 10.1002/mbo3.31

**Published:** 2012-08-03

**Authors:** Nicholas R Pannunzio, Glenn M Manthey, Lauren C Liddell, Becky Xu Hua Fu, Cai M Roberts, Adam M Bailis

**Affiliations:** 1 Department of Molecular and Cellular Biology, Beckman Research Institute of the City of HopeDuarte, California, 91010, USA; 2 The Irell and Manella Graduate School of Biological Sciences, Beckman Research Institute of the City of HopeDuarte, California, 91010, USA; 3 College of Biological Sciences, University of CaliforniaDavis, California, 95616, USA

**Keywords:** Chromosomal translocations, DNA double-strand breaks, homologous recombination, *Saccharomyces cerevisiae*, single-strand annealing

## Abstract

Homologous recombination among repetitive sequences is an important mode of DNA repair in eukaryotes following acute radiation exposure. We have developed an assay in *Saccharomyces cerevisiae* that models how multiple DNA double-strand breaks form chromosomal translocations by a nonconservative homologous recombination mechanism, single-strand annealing, and identified the Rad52 paralog, Rad59, as an important factor. We show through genetic and molecular analyses that Rad59 possesses distinct Rad52-dependent and -independent functions, and that Rad59 plays a critical role in the localization of Rad52 to double-strand breaks. Our analysis further suggests that Rad52 and Rad59 act in multiple, sequential processes that determine genome structure following acute exposure to DNA damaging agents.

## Introduction

Exposure to high doses of ionizing radiation (IR) or chemotherapeutics creates the potential for genome rearrangement by repair of DNA double-strand breaks (DSBs) (Muller et al. 2005; Argueso et al. 2008; Helleday 2010). DSBs are lethal (Resnick and Martin 1976) and organisms have evolved numerous mechanisms to repair them, most of which can be categorized into those that require homology and those that require little to no homology (Paques and Haber 1999; Symington 2002; Weinstock et al. 2006). Each mechanism uses an array of proteins to repair breaks, although what drives mechanism choice is poorly understood.

Studies have shown that homology-directed repair is subdivided into multiple pathways, two of which are gene conversion (GC) (Fogel and Mortimer 1969; Szostak et al. 1983; Nassif et al. 1994) and single-strand annealing (SSA) (Lin et al. 1984; Fishman-Lobell and Haber 1992). GC involves conservative interaction between one broken and one intact recombination partner, usually leaving the unbroken partner unchanged. SSA typically involves interactions between broken chromosome ends and leads to nonconservative genome rearrangements. SSA involving repetitive sequences on the same chromosome can result in chromosomal deletions following spontaneous or artificially induced DSBs occurring between the repeats (McDonald and Rothstein 1994; Maines et al. 1998; Smith and Rothstein 1999). SSA also generates chromosomal translocations when DSBs occur adjacent to repetitive sequences on nonhomologous chromosomes (Haber and Leung 1996; Weinstock et al. 2006; Pannunzio et al. 2008). Given the high frequency and broad distribution of repetitive elements (Kim et al. 1998; Li et al. 2001; Stenger et al. 2001) and segmental duplications (Zhang et al. 2009), substrates for SSA abound in eukaryotic genomes, and numerous studies show that both intra- and inter-chromosomal SSA events involving these sequences readily occur (Haber and Leung 1996; Fasullo et al. 1998; Richardson and Jasin 2000; Argueso et al. 2008; Pannunzio et al. 2010).

Investigation into the genetic control of SSA in budding yeast has revealed that Rad59 is a crucial factor in this mechanism (Bai and Symington 1996; Petukhova et al. 1999; Davis and Symington 2001; Pannunzio et al. 2008). Since its discovery (Bai and Symington 1996), several studies have examined the biochemical and genetic properties of Rad59. Rad59 and Rad52 share significant homology (Feng et al. 2007) and can bind and anneal ssDNA (Petukhova et al. 1999; Davis and Symington 2001). However, these two proteins have been shown to act in a sufficiently distinct manner to justify calling Rad59 a paralog of Rad52 (Wu et al. 2006). A null allele of *RAD59* displays subtle epistasis interactions with mutated alleles of *RAD52* in a variety of DSB repair events, suggesting that *RAD52* and *RAD59* cooperate in multiple contexts (Feng et al. 2007; Manthey and Bailis 2010).

Previously, our laboratory created a series of *rad59* missense alleles as tools to better understand the role Rad59 has in SSA (Pannunzio et al. 2010). Because of the genetic interactions observed, we suspected that two of these, *rad59-Y92A* and *rad59-K166A*, were separation of function mutations. Here, we further explore this possibility and report that these two alleles displayed either *RAD52*-dependent or -independent effects with respect to translocation frequency, consistent with Rad59 performing multiple, distinct functions in SSA. Molecular analyses of the activities of wild-type and mutant Rad59 proteins revealed that these functions do not require detectable association with Rad52, or the DNA sequences undergoing recombination, but can be distinguished by their effects on the association of Rad52 with DNA. Our evidence suggests that Rad59 facilitates the function of Rad52 during the repair of broken chromosomes in a manner that affects the balance between conservative and nonconservative mechanisms.

## Experimental Procedures

### Strains and plasmids

All strains used in this study were isogenic. Standard techniques for growth and genetic manipulation of yeast were used (Sherman et al. 1986). Construction of the *rad51Δ*, *rad52Δ*, *rad59Δ*, *rad59-Y92A*, *rad59-K166A*, *RAD52-FLAG*, *RAD59-FLAG*, *rad59-Y92A-FLAG*, and *rad59-K166A-FLAG* alleles have been described previously (Schild et al. 1983a,b; Pannunzio et al. 2008, 2010). The *rad59-Y92A* and *rad59-K166A* alleles were followed in crosses by allele-specific polymerase chain reaction (PCR) as described previously (Pannunzio et al. 2010).

A plasmid carrying the *RAD59-V5* fusion gene, pRS416-RAD59-V5 was the kind gift of Lorraine Symington (Davis and Symington 2001). This plasmid was manipulated to carry the *rad59-Y92A* and *rad59-K166A* alleles by swapping restriction fragments carrying the mutations for those containing the corresponding wild-type sequences.

### Determination of translocation frequency

HO endonuclease-stimulated translocation frequencies were determined selectively and nonselectively as previously described (Pannunzio et al. 2008; Liddell et al. 2011). Median translocation frequencies were determined from a minimum of 10 trials and 95% confidence intervals calculated using Prism (GraphPad, San Diego, CA). The Mann–Whitney test was used to assess statistical significance.

### Genomic Southern blot analysis

Genomic DNA was prepared from selected His^+^ and His^−^ survivors from each independent trial as described previously (Hoffman and Winston 1987). DNA was digested with *Bam*HI restriction endonuclease, separated on a 0.7% agarose, Tris-borate, ethylenediaminetetraacetic acid (EDTA) gel, and transferred to a nylon membrane (Hybond N, GE Healthcare, Pittsburgh, PA). Blots were probed with a 1.8-kb *Bam*HI genomic clone of the *HIS3* gene labeled with ^32^P by random priming (Amersham Biosciences, Piscataway, NJ). DNA fragments were visualized by autoradiography.

### Chromosome blot analysis

Chromosomes from selected His^+^ and His^−^ colonies were prepared in agarose plugs using an established protocol (Iadonato and Gnirke 1996). Chromosomes were separated on 1% agarose gels with a Bio-Rad CHEF-DR II apparatus (BioRad, Hercules, CA) as described previously (Pannunzio et al. 2008; Liddell et al. 2011). Separated chromosomes were visualized by staining with ethidium bromide, transferred to a nylon membrane, probed with the ^32^P-labeled *HIS3* genomic clone, and visualized by autoradiography.

### Determination of ectopic GC frequency

DSB-stimulated ectopic GC (EGC) between *sam1* genes in diploid strains was assayed as described previously (Pannunzio et al. 2010). Frequency of EGC was determined by dividing the number of AdoMet prototrophic recombinants by the number of viable cells plated. Median EGC frequencies from at least 10 independent cultures were determined for each genotype, 95% confidence intervals determined, and statistical significance assessed by the Mann–Whitney test.

### Coimmunoprecipitation

Coimmunoprecipitation was performed with haploid strains expressing Rad52-FLAG and Rad59-FLAG from fusion genes at the *RAD52* and *RAD59* loci. Rad59-V5 was expressed from a fusion gene driven by the *RAD59* promoter located on the single-copy plasmid pRS416-RAD59-V5 (Davis and Symington 2001). This plasmid was used to construct vectors for the expression of Rad59-Y92A-V5 and Rad59-K166A-V5. Single colonies of cells containing the appropriate combination of tagged alleles were used to inoculate 5 mL cultures of synthetic complete medium lacking uracil. Cultures were grown overnight at 30°C and used to inoculate 45 mL cultures of YPD that were grown at 30°C until mid-log phase. Cells were pelleted by centrifugation, washed with PBS, resuspended in lysis buffer and an equal volume of glass beads, and lysed at 4°C. Lysates were clarified by centrifugation before addition of protein G agarose beads and incubated at 4°C. Aliquots of precleared lysate were mixed with either anti-FLAG M2 antibody (Sigma-Aldrich, St. Louis, MO) or anti-V5 (Abcam, Cambridge, MA) and incubated at 4°C. Protein G beads were added with further incubation at 4°C, washed with lysis buffer, resuspended in sample buffer, boiled, and the suspension clarified by centrifugation. Aliquots of supernatant were loaded onto NuPAGE (polyacrylamide gel electrophoresis [PAGE]) 4-12% Bis-Tris gels and run with 2-(*N*-morpholino)ethanesulfonic acid (MES) running buffer in an XCell SureLock Mini-Cell (Invitrogen, Carlsbad, CA). Western blotting using either anti-FLAG or anti-V5 antibodies was performed as described previously (Pannunzio et al. 2010).

### Chromatin immunoprecipitation

Chromatin immunoprecipitation (ChIP) analysis was performed using a protocol defined in the Millipore EZ Chip™ kit and optimized for *Saccharomyces cerevisiae* as described previously (Meyer and Bailis 2008), with the following modifications. Haploid strains with *MATa::LEU2* at the *MAT* locus, *his3-Δ3′* at the *HIS3* locus, *his3-Δ5′* at the *LEU2* locus, and the galactose-inducible HO endonuclease coding sequence at the *TRP1* locus were used. Anti-FLAG M2 antibody was used for immunoprecipitation, and Protein G agarose beads (Pierce, Rockford, IL) were used to collect anti-FLAG bound protein/DNA complexes. DNA fragments were recovered and analyzed by PCR. Detection of DNA fragments from genomic sequences near the DSB at the *HIS3* locus was accomplished using the HISChIP 5′ (5′-AGA GCG GTG GTA GAT CTT TCG-3′) and HISChIP 3′ (5′-TTG CCT CGC AGA CAA TCA ACG-3′) primers, which created a 150-bp product. As a control, we confirmed that no product was generated from IPs lacking antibody. Normalization was achieved using signals obtained by amplifying a 200-bp region of the *SAM1* locus using the SAMChIP 5′ (5′-GCC CTT GCC TAC TAG TGC ATT T-3′) and SAMChIP 3′ (5′-CGA AGC TAA CCG AAA AAC AAC G-3′) primers. Reactions were run for 25–30 cycles, depending on the amount of signal produced, so that amplification was kept in the linear range for image analysis. PCR products were resolved on 3% agarose gels, stained with ethidium bromide, and imaged using a Typhoon 8600. Images were quantitated using the ImageQuant 5.0 software package.

### Western blot analysis

Western blot analysis was performed using established methods with minor modifications (Sambrook and Russell 2001). Twenty-milliliter cultures were grown at 30°C to mid log and harvested via centrifugation at 5000 RPM. Cells were washed with 0.5 mL H_2_O and transferred to a fresh Eppendorf tube. Cell pellets were washed once with 20% trichloroacetic acid (TCA), resuspended in 0.200 mL of 20% TCA and 0.2 mL glass beads, and the cells disrupted by vortexing at room temperature. Extracts were transferred to fresh Eppendorf tubes, and the beads washed twice with 0.2 mL 20% TCA and added to the extracts. Pools were centrifuged for 10 min at 3000 RPM, and pellets resuspended in 100 *μ*L of 2× loading buffer (166 mmol/L Tris-HCl, pH 8.0, 53 mmol/L Tris-Base, 26.6% glycerol, 5.3% sodium dodecyl sulfate [SDS], 0.007% bromophenol blue), 80 *μ*L 1 mol/L Tris-HCl, and 20 *μ*L 500 mmol/L Tris (2-carboxyethyl) phosphine hydrochloride (TCEP). Samples were boiled and clarified by centrifugation at 3000 RPM. Five microliters of each sample was resolved on a NuPAGE 4–12% Bis-Tris gel with MES running buffer in an XCell SureLock Mini-Cell. Protein was transferred to an Immobolin-P membrane (Millipore, Billerica, MA) using a Mini Trans-Blot Electrophoretic Transfer Cell (BioRad). The membrane was blocked in 5% milk and then probed with 1:2000 dilutions of the primary antibodies, ANTI-FLAG M2 (Sigma-Aldrich) and anti-GAPDH/Clone GA1R (Aviva Systems Biology, San Diego, CA) at room temperature. After washing, the membrane was probed with a 1:10,000 dilution of the secondary antibody, goat anti-mouse HRP (Thermo Scientific, Rockford, IL) at room temperature. A Pierce SuperSignal West Femto Kit (Thermo Scientific) was used to produce a chemiluminescence signal, which was detected using Kodak BioMax XAR film.

## Results

### Translocation formation by HR rescues broken chromosomes

**Figure 1:**
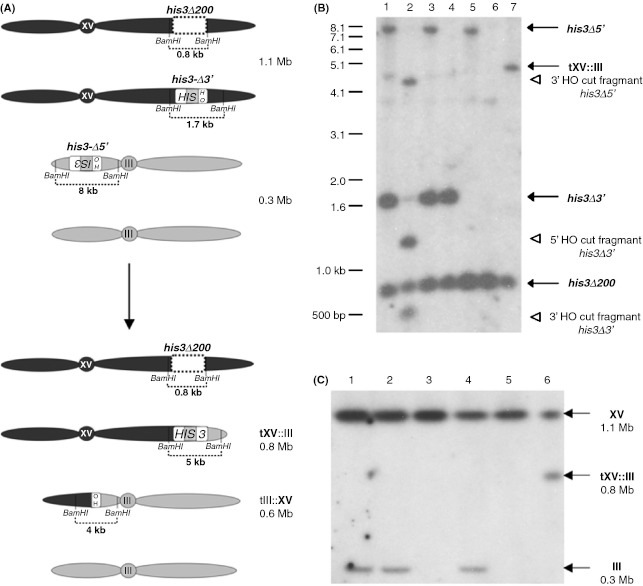
The T2 system produces a tXV:III chromosome following creation of two DSBs in diploid cells. (A) The T2 system – A 3′ truncated allele of *HIS3* (*his3-Δ3*′) is located at the *HIS3* locus on one copy of chromosome XV (black) and a 5′ truncated allele (*his3-Δ5′*) is at the *LEU2* locus on one copy of chromosome III (gray). Chromosome sizes are indicated in megabases. The *his3* substrates share 311 bp of identical sequence, indicated by dark gray boxes. Adjacent to each substrate is a 117-bp fragment of the *MAT* locus containing the recognition sequence for the HO endonuclease. The *his3-Δ200* allele at the *HIS3* locus on the other copy of chromosome XV (white box with dashed outline) is missing sufficient information to contribute to the generation of an intact *HIS3* gene by HR. Upon addition of galactose, HO endonuclease is expressed and DSBs are formed adjacent to each *his3* substrate. Repair of the DSBs by SSA creates a tXV:III translocation product that is 0.8 Mb in length and carries a functional *HIS3* gene. The reciprocal tIII:XV translocation product is 0.6 Mb in length. Sizes of fragments generated by digestion of genomic DNA with *Bam*HI from cells either prior to or following galactose induction and probing with a ^32^P-labeled 1.8-kb *Bam*HI genomic clone carrying the *HIS3* gene are indicated. (B) Blots of genomic DNA from cells before, during, and after recovery from DSB formation by HO endonuclease – genomic DNA was digested with *Bam*HI endonuclease, resolved on a 0.7% agarose gel, blotted to nylon, probed with a ^32^P-labeled 1.8-kb *HIS3* genomic clone, and autoradiographed as described in the Experimental Procedures. Locations of molecular weight markers are indicated on the left side of the figure and are marked in kilobase pairs. Identities of the species on the blot are indicated on the right side of the figure. Lanes: (1) parent strain, (2) parent strain following 1 h of *GAL::HO* expression, (3) class 1 His^−^ survivor, (4) class 2 His^−^ survivor, (5) class 3 His^−^ survivor, (6) class 4 His^−^ survivor, (7) His^+^ survivor (see Fig. S1). (C) Blots of chromosomes from cells before and after recovery from DSB formation – chromosomes were prepared in agarose plugs, run on a 1% agarose gel, blotted to nylon, probed with the ^32^P-labeled 1.8-kb *HIS3* genomic clone, and autoradiographed as described in the Experimental Procedures. Identities of the species on the blot are indicated on the right side of the figure. Lanes: (1) parent strain, (2) class 1 His^−^ survivor, (3) class 2 His^−^ survivor, (4) class 3 His^−^ survivor, (5) class 4 His^−^ survivor, (6) His^+^ survivor.

Recently, our work has explored the formation of chromosomal translocations by homologous recombination (HR) in diploid *S. cerevisiae* cells (Manthey et al. 2009; Manthey and Bailis 2010). Our assays measure HR between 311-bp sequences shared by the *his3-Δ3′* substrate at the *HIS3* locus on one copy of chromosome XV and the *his3-Δ5′* substrate inserted at the *LEU2* locus on one copy of chromosome III (Fig. 1A). A *his3-Δ200* allele that lacks sufficient *HIS3* sequence to generate an intact *HIS3* gene by HR is located at the *HIS3* locus on the other copy of chromosome XV. The mating-type loci on both copies of chromosome III are ablated to prevent DSBs there following expression of a galactose-inducible HO endonuclease gene at the *TRP1* locus on chromosome V. Assays are modified to measure translocation formation that occurs spontaneously (T0), or following HO-catalyzed DSBs adjacent to one (T1) or both (T2) substrates (Pannunzio et al. 2008; Manthey and Bailis 2010). The T2 assay simulates a level of IR damage sufficient to create DSBs adjacent to multiple repetitive sequences in the yeast genome. Such conditions result in chromosomal translocations by HR at a high frequency (Argueso et al. 2008).

T2 translocation frequencies that measure the concomitant formation of an intact *HIS3* gene and a tXV:III translocation chromosome were determined in wild-type cells under conditions that select for His^+^ recombinants either directly on histidine-less medium (2.73 × 10^−2^) or indirectly by first plating to nonselective medium and replica plating to histidine-less medium (2.57 × 10^−2^), yielding similar results (*P* = 0.79)(Fig. S1A). The reciprocal tIII:XV translocation formed by annealing and ligating complementary 4-bp overhangs generated by HO cleavage on the two remaining chromosome fragments is observed on genomic Southern and chromosome blots of some His^+^ recombinants but is not selectable (Pannunzio et al. 2008).

Additionally, 20 His^+^ and 20 His^−^ survivors from independent cultures were examined by genomic Southern and chromosome blotting to document both selectable and nonselectable outcomes of the T2 assay (Fig. 1B and C). All His^+^ survivors examined possessed a 5.0-kb *Bam*HI fragment indicative of an intact *HIS3* gene on a 0.8 Mb tXV:III translocation chromosome (Fig. S1B). Also, all of the His^+^ recombinants lacked the 1.7-kb *Bam*HI *his3-Δ3′* substrate fragment, and the 7.8-kb *Bam*HI fragment and 0.3 Mb chromosome III signal indicative of the *his3-Δ5′* substrate, demonstrating the nonconservative nature of the repair process. Evidence of the tIII:XV reciprocal translocation chromosome observed previously in a minority of His^+^ survivors was not observed among this set of recombinants (Pannunzio et al. 2010, 2008; Manthey et al. 2009; Manthey and Bailis 2010; Liddell et al. 2011).

In contrast to the His^+^ survivors, 13 of the 20 independent His^−^ survivors possessed both the *his3-Δ3′* and *his3-Δ5′* substrates (Class 1) (Figs. 1B and S1C). These substrates maintained intact HO cut site sequences as colonies yielded His^+^ papillae after replica plating onto galactose-containing medium lacking histidine (L. Liddell and A. Bailis, unpubl. results). As uncut *his3* substrates are nearly undetectable on blots after expression of HO (Fig. 1B), intact substrate chromosomes in survivors most likely indicate perfect rejoining by nonhomologous end-joining (NHEJ). Four of the His^−^ survivors possessed the *his3-Δ3′* substrate alone (Class 2), one possessed *his3-Δ5′* alone (Class 3), and two displayed no evidence of either substrate (Class 4). Loss of the substrates in these survivors could be due to HR events with the *LEU2* and *HIS3* loci on the copies of chromosomes III and XV not carrying the substrates, NHEJ events that occur after extensive processing to eliminate the substrates, or chromosome loss.

Our analysis of the His^+^ and His^−^ survivors suggests that wild-type cells possess a finite capacity to repair DSBs by both HR- and NHEJ-related processes. Plating efficiencies before and after DSB formation were not significantly different (*P* = 0.22), indicating that whichever mechanism is engaged to repair DSBs, there is a minimal effect on viability (Table S1). As we have demonstrated previously (Pannunzio et al. 2008), loss of *DNL4*, which encodes the DNA ligase required for NHEJ (Wilson et al. 1997), had no significant effect on formation of the His^+^ recombinants obtained either selectively or nonselectively (Fig. S1A). Together, these results suggest that in this assay NHEJ and SSA do not compete for the rescue of the broken chromosomes.

### *RAD59* has distinct *RAD52*-dependent and -independent roles in translocation formation by SSA

**Figure 2:**
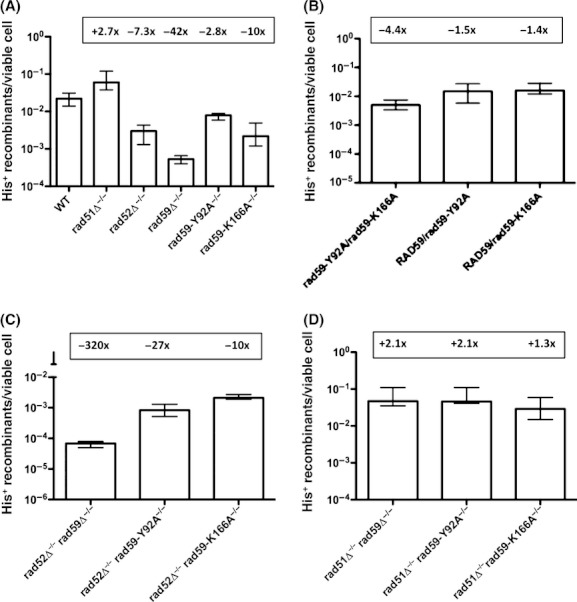
T2 translocation frequencies in homozygous wild-type and HR defective diploids. Translocation frequencies were determined selectively as described in the Experimental Procedures. Median frequencies and 95% confidence intervals from at least 10 independent trials are displayed. Fold decreases (−) and increases (+) from wild-type are indicated. (A) Frequencies of His^+^ colony formation in wild-type and single mutant diploids. (B) Dominance/recessiveness of *rad59* mutant alleles. (C) Epistasis analysis with *rad52Δ* and *rad59* mutant alleles. (D) Epistasis analysis with *rad51Δ* and *rad59* mutant alleles.

Genetic analysis revealed that the tXV:III translocation chromosome is generated by SSA with *RAD59* playing a dominant role (Pannunzio et al. 2008). Here, using the T2 assay, we again confirm the necessity of *RAD59* as the translocation frequency in the *rad59Δ*^*−/−*^ homozygote is 42-fold reduced from the wild-type frequency (Fig. 2A). As previously reported (Manthey and Bailis 2010), the translocation frequency in the *rad52Δ*^*−/−*^ homozygote is reduced only 7.3-fold, indicating that *RAD59* is at least as important to the process as *RAD52*.

The role of *RAD59* was further elucidated by studying missense alleles that exhibit various effects on formation of translocations and interstitial deletions (Pannunzio et al. 2010). Two of these alleles, *rad59-Y92A* and *rad59-K166A,* confer distinct translocation frequencies. This pattern was recapitulated here with the *rad59-Y92A*^*−/−*^ homozygote displaying a statistically significant (*P* = 0.011) 2.8-fold reduction in translocation frequency and the *rad59-K166A*^*−/−*^ homozygote displaying a significantly greater (*P* = 0.001) 10-fold reduction (Fig. 2A).

Both mutant alleles were found to be recessive as the 1.5-fold and 1.4-fold reduced frequencies of translocation in the *RAD59/rad59-Y92A* and *RAD59/rad59-K166A* heterozygotes were not significantly different from wild-type (*P* = 0.20 and 0.49), but were significantly different from their respective mutant homozygotes (*P* = 0.043 and 0.0001) (Fig. 2B). Interestingly, the *rad59-Y92A/rad59-K166A* heterozygote displayed a 4.4-fold reduced frequency of translocation that was intermediate to the frequencies observed in the *rad59-Y92A*^*−/−*^ (*P* = 0.019) and *rad59-K166A*^*−/−*^ (*P* = 0.0084) homozygotes. This codominance suggests that the products of both alleles together contribute to translocation formation.

Previous work also showed that the translocation frequency in *rad52Δ*^*−/−*^ *rad59Δ*^*−/−*^ double homozygotes is synergistically reduced relative to the frequencies observed in *rad52Δ*^*−/−*^ and *rad59Δ*^*−/−*^ single homozygotes (Pannunzio et al. 2008). Similar to those results, the T2 translocation frequency in the *rad52Δ*^*−/−*^ *rad59Δ*^*−/−*^ double homozygote was reduced 324-fold, which is significantly lower (*P* ≤ 0.0001) than the frequencies observed in either the *rad52Δ*^*−/−*^ or *rad59Δ*^*−/−*^ single homozygotes (Fig. 2C). Plating efficiencies before and after DSB formation in the strains examined were similar for the single homozygotes, and while plating efficiencies decreased approximately twofold after DSB formation in the double homozygote, the synergistic effect on translocation frequency is unlikely to be due to changes in the ability to survive DSBs (Table S1). Therefore, *RAD52* and *RAD59* act as paralogs as they make distinct contributions to translocation formation by SSA (Wu et al. 2006; Feng et al. 2007).

Epistasis relationships between the *rad59* missense alleles and *rad52Δ* were also examined. The translocation frequency in the *rad52Δ*^*−/−*^ *rad59-K166A*^*−/−*^ double homozygote was 10-fold lower than wild-type and was not significantly different from *rad52Δ*^*−/−*^ (*P* = 0.44) or *rad59-K166A*^*−/−*^ (*P* = 0.79) (Fig. 2A and C). This mutually epistatic relationship suggests that *rad59-K166A* confers a defect in a *RAD52*-dependent function of *RAD59*. In contrast, the *rad59-Y92A* allele displayed a synergistic relationship with *rad52Δ* as the translocation frequency in the *rad52Δ*^*−/−*^ *rad59-Y92A*^*−/−*^ double homozygote was 27-fold lower than wild-type and significantly lower than the frequencies observed in *rad52Δ*^*−/−*^ (*P* = 0.0032) and *rad59-Y92A*^*−/−*^ (*P* ≤ 0.0001). This suggests that *rad59-Y92A* confers a defect in a function of *RAD59* independent from that of *RAD52*. These data demonstrate that Rad59 has genetically separable *RAD52*-dependent and -independent functions.

Recent studies have indicated that *RAD59* is only required for translocation formation by SSA if Rad51 nucleoprotein filaments form at the DSBs (Manthey and Bailis 2010). This was supported by the observation that translocation frequency was 2.1-fold higher than wild-type in a *rad51Δ*^*−/−*^ *rad59Δ*^*−/−*^ double homozygote, which was not statistically different from the 2.7-fold increase observed in the *rad51Δ*^*−/−*^ single homozygote (*P* = 0.50)(Fig. 2A and D). This indicates that translocation formation is under distinct genetic control in the absence of *RAD51*.

Epistasis interactions of *rad59-Y92A* and *rad59-K166A* with *rad51Δ* were also examined (Fig. 2D). Neither the 2.1-fold increased translocation frequency in the *rad51Δ*^*−/−*^ *rad59-Y92A*^−/−^ double homozygote nor the 1.3-fold increased frequency in the *rad51Δ*^*−/−*^ *rad59-K166A*^−/−^ double homozygote was significantly different from that of *rad51Δ*^*−/−*^ (*P* = 0.91 and 0.35) (Fig. 2A and D). These results suggest that the *RAD52*-dependent and -independent roles of *RAD59* are unimportant for translocation formation by SSA in the absence of *RAD51*.

### Mutations in *RAD59* affect EGC

The epistasis relationships of the *rad59* alleles with *rad52Δ* in the T2 assay suggested that they might confer distinct effects in other *RAD52*-dependent DSB repair assays. Therefore, we measured the frequency of EGC following creation of an HO-catalyzed DSB between the *sam1-ΔBglII-HOcs* allele at the *SAM1* locus on one copy of chromosome XII and an unbroken *sam1-ΔSalI* allele at the *HIS3* locus on one copy of chromosome XV. Repair results in a functional *SAM1* gene and *S*-adenosylmethionine prototrophy (Manthey and Bailis 2010; Pannunzio et al. 2010).

The 1487- and 3056-fold reduced frequencies measured in the *rad51Δ*^*−/−*^ and the *rad52Δ*^*−/−*^ homozygotes, respectively, indicated that both *RAD51* and *RAD52* are critically important for EGC (Table 1). In contrast, the frequency measured in the *rad59Δ*^−/−^ homozygote was not significantly different from wild-type (*P* = 0.14) suggesting that *RAD59* is not required for EGC. However, the *rad59-Y92A*^*−/−*^ homozygote displayed a modest, but significant 3.1-fold decrease in EGC (*P* = 0.034), while the *rad59-K166A*^*−/−*^ homozygote displayed a significant 4.2-fold increase (*P* = 0.0007). These results suggest that Rad59 is a minor factor in *RAD51*- and *RAD52*-dependent GC.

**Table 1:** DSB-stimulated ectopic gene conversion in wild-type and homozygous mutant diploid strains

Genotype	Frequency (Sam^+^ recombinants/survivor)^a^
Wild-type	1.1 × 10^−3^ (0.6, 1.6)
*rad51Δ*^*−/−*^	7.4 × 10^−7^ (4.0, 11.8) [−1487]
*rad52Δ*^*−/−*^	3.6 × 10^−7^ (1.6, 5.6) [−3056]
*rad59Δ*^*−/−*^	6.7 × 10^−4^ (3.7, 11.2) [−1.6]
*rad59-Y92A*^*−/−*^	3.6 × 10^−4^ (1.9, 5.8) [−3.1]
*rad59-K166A*^*−/−*^	4.6 × 10^−3^ (1.6, 3.8) [+4.2]

a The median frequency of EGC is reported for each strain from a minimum of 10 independent determinations as described in the Experimental Procedures. The 95% confidence intervals are in parentheses. The fold differences from (+ = greater than, − = less than) wild-type are in brackets.

### Mutations in *RAD59* affect interaction between Rad59 and Rad52

Rad59 works in concert with Rad52 in vitro (Wu et al. 2006, 2008). As the *rad59-K166A* and *rad59-Y92A* alleles differ with respect to their genetic interactions with *rad52Δ*, we investigated their effects on the physical interaction between Rad52 and Rad59 using coimmunoprecipitation (co-IP). Fusion genes that encode C-terminally FLAG-tagged Rad52 and either FLAG- or V5-tagged wild-type and mutated Rad59 were used (Davis and Symington 2001; Meyer and Bailis 2008). *RAD52-* and *RAD59-FLAG* alleles were located at the native *RAD52* and *RAD59* loci with expression driven from their native promoters, while the *RAD59-V5* alleles were located on centromere-containing plasmids with expression driven from the *RAD59* promoter.

**Figure 3:**
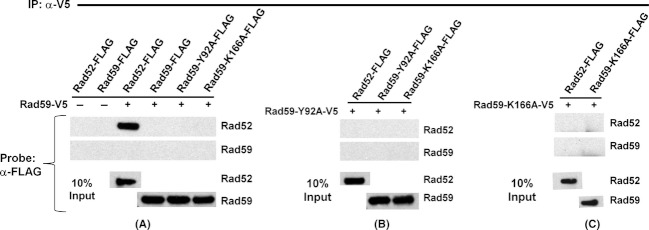
Wild-type Rad59, but not Rad59-Y92A or Rad59-K166A, interacts with Rad52. Proteins were precipitated from yeast whole cell extracts with anti-V5 antibody. Precipitated proteins where separated on SDS-PAGE gels, transferred to a PVDF membrane, and probed with anti-FLAG antibody. Each blot displays the results from one of at least three independent experiments. (A) Strains expressed wild-type Rad59 with a C-terminal fusion of the V5 epitope (except for lanes 1 and 2, which are included to demonstrate that no FLAG-tagged proteins are present in the anti-V5 IP without the presence of a V5-tagged protein). Above each lane is indicated which C-terminal FLAG-tagged Rad52 or Rad59 proteins were coexpressed in the strain. The lower blot displays the Rad52-FLAG and Rad59-FLAG signals generated by probing 10% of the whole cell extracts prior to immunoprecipitation with anti-FLAG antibody. (B) Same as in (A) except that each strain expressed the V5-tagged Rad59-Y92A mutant protein. (C) Same as in (A) except that each strain expressed the V5-tagged Rad59-K166A mutant protein.

Similar to previous studies (Davis and Symington 2001, 2003), we observed interaction between wild-type Rad59 and Rad52 as Rad59-V5 complexes immunoprecipitated from whole cell extracts, and probed with anti-FLAG produced a Rad52-FLAG signal (Fig. 3A). No signal was observed in controls where immunoprecipitation was performed in cells producing FLAG-tagged Rad52 or Rad59 but lacking a V5-tagged Rad59. Unlike a previous study (Davis and Symington 2003), we did not observe interaction between wild-type Rad59 proteins (Fig. 3A). Notably, that study was conducted under conditions of overexpression, which may promote self-association.

Interestingly, while the *rad59-Y92A* and *rad59-K166A* mutations had distinct effects on HR, they both led to undetectable levels of binding to Rad52 (Fig. 3B and C). As with wild-type Rad59 proteins, no combination of wild-type or mutant Rad59 proteins was observed to interact. Identical results were obtained when proteins were immunoprecipitated with anti-FLAG and probed with anti-V5 (Fig. S2). Previously, we have shown that the mutated Rad59 proteins are present at levels comparable to wild-type Rad59, so the lack of interaction is not an issue of protein stability (Pannunzio et al. 2010). Instead, these results indicate that the *rad59-Y92A* and *rad59-K166A* alleles encode proteins whose association with Rad52 is below the level of detection. This suggests that direct interaction between Rad59 and Rad52 may not be required for Rad59 function in translocation formation and EGC (Fig. 2 and Table 1).

### Rad59 influences association of Rad52 with DSBs

**Figure 4:**
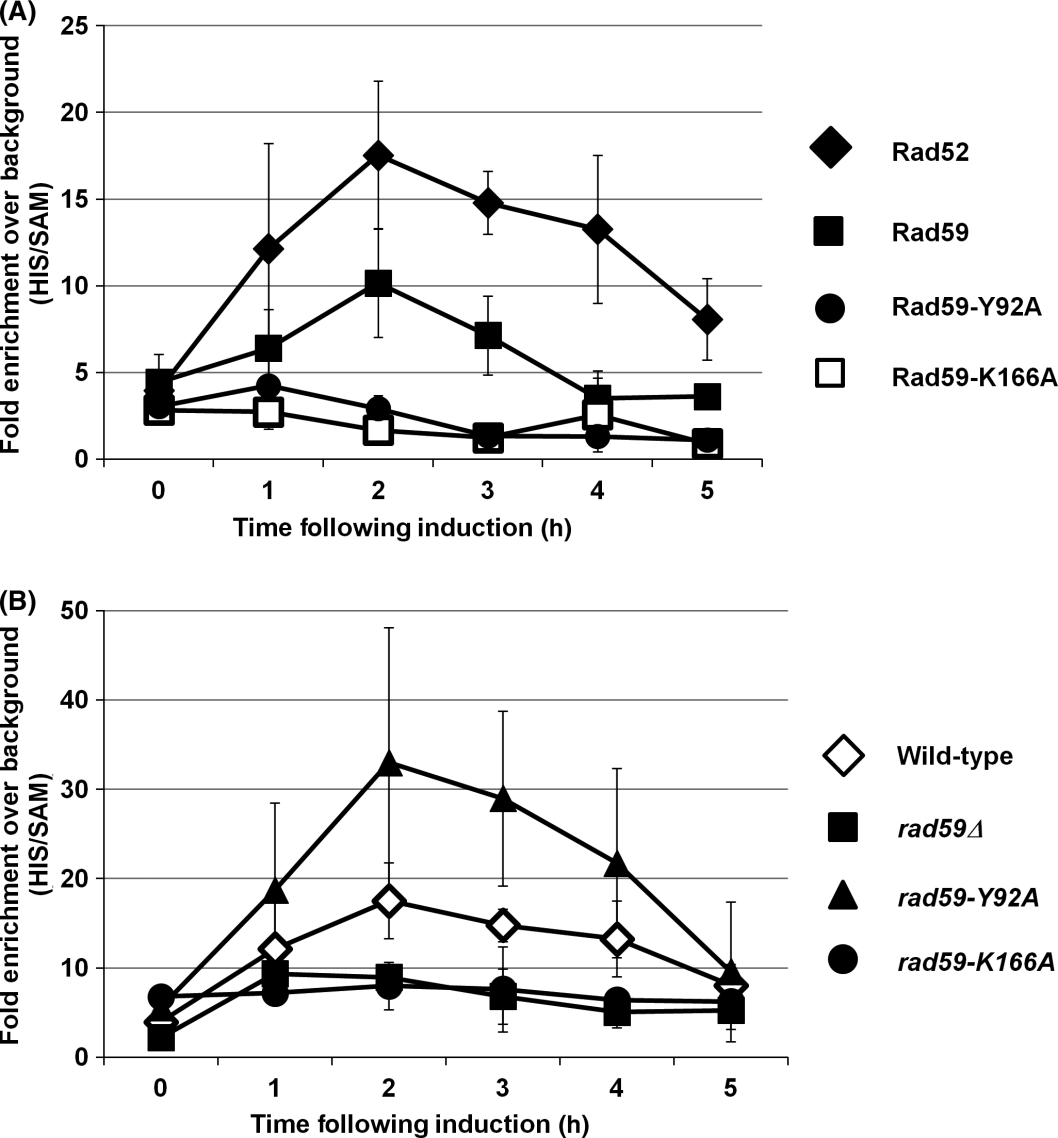
The *rad59Δ* and *rad59-K166A* mutations prevent significant accumulation of Rad52 at DSBs. Cells expressing the indicated FLAG-tagged proteins from their endogenous chromosomal loci were collected before (0 h), and at specific timepoints after the induction of HO endonuclease and subjected to ChIP using anti-FLAG antibody as described in the Experimental Procedures. Immunoprecipitated DNA was used as the template for multiplex PCR reactions using primers complementary to sequences adjacent to the *his3-Δ3′* substrate at the *HIS3* locus on chromosome XV, and the *SAM1* coding sequence on chromosome XII. Products were run on 3% agarose gels, stained with ethidium bromide, and the fluorescent signals quantified. ChIP signals for each timepoint were determined by dividing the *HIS3* signal by the *SAM1* signal. Mean fold enrichment ratios and 95% confidence intervals were determined from at least three independent trials and were plotted against time after the induction of HO endonuclease. (A) Fold enrichment of each of the FLAG-tagged proteins indicated in the legend at the *HIS3* locus relative to the *SAM1* locus during the time course. (B) Fold enrichment of FLAG-tagged Rad52 at the *HIS3* locus relative to the *SAM1* locus during the time course in the wild-type and *rad59* mutant strains indicated in the legend.

ChIP was used to demonstrate the association of Rad52 with a DSB during translocation formation by SSA (Meyer and Bailis 2008). Similar to previous experiments, we observed a peak, 4.4-fold enrichment of Rad52 at the *his3-Δ3′* substrate 2 h after DSB formation, followed by progressive diminishment of signal (Fig. 4A). Similarly, a peak, 2.2-fold enrichment of Rad59 was observed at 2 h that diminished at least as quickly. In contrast, Rad59-Y92A and Rad59-K166A proteins were not enriched at DSBs above background levels, indicating diminished binding of Rad59 to DNA or, perhaps, to Rad52.

Investigation into the biochemical properties of Rad52 in vitro showed that Rad59 augments the ability of Rad52 to anneal complementary single-stranded DNA strands in the presence of Rad51 filaments (Wu et al. 2008), suggesting that mutations in *RAD59* may affect association of Rad52 with the recombination substrates. Accordingly, in a *rad59Δ* mutant, Rad52 did not associate with the DSB above background levels (Fig. 4B). The *rad59-K166A* allele also led to reduced Rad52 DSB association, consistent with Rad59 having lost the ability to support Rad52. Rad52 protein levels were not reduced in *rad59Δ* and *rad59-K166A* mutant cells, indicating that failure to observe association of Rad52 with the DSB was not due to a decrease in the amount or stability of Rad52 (Fig. S3).

Strikingly, the *rad59-Y92A* allele conferred levels of Rad52 association that were not significantly different from the wild-type (*P* > 0.07 at all time points), indicating that the ability of Rad59 to support Rad52 was unaffected. This result was particularly interesting as *rad59-Y92A* disabled the interaction between Rad59 and Rad52 detectable by Co-IP, and suggests that Rad59 may affect association between Rad52 and the DSB indirectly. This also defines *rad59-Y92A* and *rad59-K166A* as separation of function alleles at the molecular level.

Data indicating that Rad59 can affect the accumulation of Rad52 at a translocation substrate after DSB formation contrasts with a previous report where fluorescently tagged Rad52 was observed to form nuclear foci in *rad59*-null mutant cells following exposure to IR (Lisby et al. 2004). While these differences are intriguing, it is important to note that the previous study documented responses to the multiple, chemically diverse lesions that result from radiation exposure, while the current study examined events at a single, defined DSB. Furthermore, the requirements for a protein to form a cross-link with a specific DNA sequence, as in ChIP analysis, are likely to be different from those necessary for the formation of a focus.

## Discussion

We have provided further evidence for the discrete involvement of *RAD59* and *RAD52* in SSA through the use of separation of function alleles that confer distinct *RAD52*-dependent and -independent defects. Examining the results of our genetic and molecular results together, it becomes clear that the mechanism of SSA exists within a network of DNA repair modalities, the relationship between which is controlled by a few key DNA repair factors. With respect to the engagement of the SSA machinery at DSBs, a critical initial condition appears to be the presence of Rad51 filaments. There is strong evidence that a Rad51-filament is inhibitory to SSA (McDonald and Rothstein 1994; Stark et al. 2004; Wu et al. 2008; Manthey and Bailis 2010). However, it appears also to be true that the presence of Rad51 filaments determines the requirement for Rad59 (Fig. 2D) and other components of the canonical SSA machinery (Manthey and Bailis 2010), at least for translocation formation by SSA. The genetic and molecular data presented here suggest the possibility that at least one of the functions of Rad59 is facilitating the replacement of the Rad51-filament by Rad52, which, in turn, facilitates the annealing of complementary single-stranded DNA strands.

Like the *rad59Δ* allele, *rad59-K166A* reduces the recruitment of Rad52 to DSBs to below detectability, but the *rad59-Y92A* mutation does not (Fig. 4B). Consequently, we suggest that *rad59-K166A* confers a defect in strand annealing, while *rad59-Y92A* results in a defect in a subsequent step. Previous genetic data indicate that this may be in nonhomologous tail removal along with Rad1-Rad10 and Msh2-Msh3 (Sugawara et al. 2000; Lyndaker and Alani 2009; Manthey et al. 2009; Pannunzio et al. 2010). Interestingly, while recruitment of Rad52 to DSBs in *rad59-Y92A*^*−/−*^ homozygotes is at least as robust as in wild-type, our data indicate that the *rad59-Y92A* mutation diminishes the interaction of Rad59 with Rad52 to below detectability (Fig. 3B). This may indicate that a strong, direct interaction between Rad59 and Rad52 is unnecessary for the initial stages of SSA, and that interactions with other proteins may be more critical. Kowalczykowski and colleagues observed a weak, direct interaction between Rad59 and Rad51 in vitro (Wu et al. 2008), suggesting that Rad59 may influence the association of Rad52 with DNA through a direct interaction with Rad51. This possibility is currently under investigation.

In summary, our data suggest that Rad52 and Rad59 play multiple, sequential roles in the response to DSBs. The first role of Rad52 is to facilitate Rad51 nucleoprotein filament formation, which it does by binding DNA, evicting the single-stranded DNA binding protein, replication protein A, from DNA ends, and binding to Rad51 (Heyer et al. 2010). This process is required for DSB repair by strand invasion-mediated mechanisms such as EGC, but inhibits repair by SSA. The second role for Rad52 is engaged if the nucleoprotein filament is not utilized for HR, whereupon Rad52 can act with Rad59, perhaps in conjunction with Srs2 (Manthey and Bailis 2010), to replace Rad51 on DNA and promote annealing with complementary sequences at other broken chromosome ends. This event is followed by the removal of nonhomologous tails created by the annealing event that is executed by the Rad1-Rad10 nuclease and coordinated by Rad59 (Lyndaker and Alani 2009). This order of events would favor the maintenance of genome integrity as strand invasion-mediated events between allelic sequences on sister chromatids and homologs conserve genome structure, while SSA-mediated events between nonallelic sequences on the same or different chromosomes are invariably nonconservative. This order of events would also explain why the original chromosome structure is so rapidly recapitulated in cultures of yeast cells following exposure to acute doses of IR, but unique, nonreciprocal translocations between delta elements are frequently observed in individual survivors (Argueso et al. 2008).

As SSA is an efficient mechanism of DSB repair in both yeast and mammalian cells (Prado and Aguilera 1995; Ivanov et al. 1996; Haber and Leung 1996; Liang et al. 1998; Richardson and Jasin 2000; Pannunzio et al. 2008), a mammalian homolog of Rad59 could have an important role in governing genome stability following high levels of DNA damage. While no homolog has been positively identified, mutating *RAD52* in mouse cells reduces the frequency of DSB repair by SSA but not GC (Stark et al. 2004), much like mutating *RAD59* in yeast (Pannunzio et al. 2010). This suggests the possibility that RAD52 functions similarly in mouse cells. As human RAD52 interacts physically and functionally with XPF-ERCC1 (Motycka et al. 2004), the human homolog of Rad1-Rad10, RAD52 may coordinate strand annealing with the removal of nonhomologous tails during SSA, much as Rad59 may do in yeast (Lyndaker and Alani 2009). If RAD52 is performing these functions in human cells, it may be a viable target for drugs to attenuate SSA events that contribute to therapy-related drug resistance and disease progression in patients with chronic myelogenous leukemia and other myeloproliferative disorders (Cramer et al. 2008; Fernandes et al. 2009).

## Supporting Information

**Figure S1.** (A) T2 translocation frequencies in wild-type and *dnl4*Δ^−/−^ mutant diploids obtained with and without selection. Frequencies of translocation formation were determined in wild-type and *dnl4*Δ^−/−^ mutant diploid strains both selectively and nonselectively as described in the Experimental Procedures. Median frequencies and 95% confidence intervals determined from at least 10 independent trials are displayed. (B) Genomic Southern blots of independent His^+^ survivors. Genomic DNA from the parent strain and from 16 independent His^+^ survivors was digested with *Bam* HI endonuclease, run on a 0.7% agarose gel, blotted to nylon, probed with a ^32^P-labeled 1.8-kb *HIS3* genomic clone, and autoradiographed as described in the Experimental Procedures. Locations of molecular weight markers are indicated on the left side of the figure and marked in kilobase pairs. Identities of the species on the blot are indicated on the right side of the figure. Lanes: (1–16) His^+^ recombinants, (P) His^−^ parent. (C) Genomic Southern blots of independent His^−^ survivors. Genomic DNA from the parent strain and 16 independent His^−^ survivors was digested with *Bam*HI endonuclease, run on a 0.7% agarose gel, blotted to nylon, probed with a ^32^P-labeled 1.8-kb *HIS3* genomic clone, and autoradiographed as described in the Experimental Procedures. Locations of molecular weight markers are indicated on the left side of the figure and are marked in kilobase pairs. Identities of the species on the blot are indicated on the right side of the figure. Lanes: (1–16) His^−^ survivors, (P) His^−^ parent.

**Figure S2.** Wild-type Rad59, but not Rad59-Y92A or Rad59-K166A, interacts with Rad52. Proteins were precipitated from yeast whole cell extracts with anti-FLAG antibody. Precipitated proteins where separated on SDS-PAGE gels, transferred to a PVDF membrane, and probed with anti-V5 antibody. Each blot displays the results from one of at least three independent experiments. (A) All strains expressed wild-type Rad59 with a C-terminal fusion of the V5 epitope as indicated on the left side of the blot. Above each lane is indicated whether each strain expressed untagged wild-type Rad52 or Rad59 proteins, FLAG-tagged wild-type Rad52, or FLAG-tagged wild-type or mutant Rad59. The lower blot displays the Rad59-V5 signals generated by probing 10% of the whole cell extracts prior to immunoprecipitation with anti-V5 antibody. (B) Same as in (A) except that each strain expressed the V5-tagged Rad59-Y92A mutant protein. (C) Same as in (A) except that each strain expressed the V5-tagged Rad59-K166A mutant protein.

**Figure S3.** Levels of Rad52 are not reduced in *rad59* mutants. Aliquots of whole cell extracts from wild-type and *rad59* mutant cells were subjected to SDS-PAGE, transferred to a PVDF membrane, and probed with anti-FLAG antibody. Each blot displays the results from one of at least three independent experiments.

**Table S1.** Plating efficiencies of wild-type and mutant diploid strains before and after HO-stimulated DSB formation on two chromosomes.
