# Supplementary material for: Rad59 regulates association of Rad52 with DNA double-strand breaks
Source: Microbiologyopen. 2012 Aug 3;1(3):285–97. doi: 10.1002/mbo3.31 (PMC3496973; doi:10.1002/mbo3.31)
Supplement: Supplementary file 1 [file mbo30001-0285-SD1.doc]

**Rad59 Regulates Association of Rad52 with DNA Double-Strand Breaks**

Nicholas R. Pannunzio^1,2,†^, Glenn M. Manthey^1^, Lauren C. Liddell^1, 2^, Becky Xu Hua Fu^3^, Cai M. Roberts^1,2^, and Adam M. Bailis^1*^

^1^Department of Molecular and Cellular Biology, Beckman Research Institute of the City of Hope, 1500 E. Duarte Road, Duarte, CA 91010, USA

^2^The Irell and Manella Graduate School of Biological Sciences, Beckman Research Institute of the City of Hope,1500 E. Duarte Road, Duarte, CA 91010, USA

^3^College of Biological Sciences, University of California, One Shields Avenue, Davis, CA 95616, USA

^*^Corresponding Author: Adam M. Bailis, Ph.D., abailis@coh.org

**Figure S1.**

**A. T2 translocation frequencies in wild-type and *dnl4∆^-/-^* mutant diploids obtained with and without selection.**

Frequencies of translocation formation were determined in wild-type and *dnl4∆*^-/-^ mutant diploid strains both selectively and non-selectively as described in the Materials and Methods. Median frequencies and 95% confidence intervals determined from at least 10 independent trials are displayed.

**B. Genomic Southern blots of independent His^+^ survivors.**

Genomic DNA from the parent strain and from 16 independent His^+^ survivors was digested with *Bam* HI endonuclease, run on a 0.7% agarose gel, blotted to nylon, probed with a ^32^P-labeled 1.8 kb *HIS3* genomic clone and autoradiographed as described in the Materials and Methods. Locations of molecular weight markers are indicated on the left side of the figure and marked in kilobase pairs. Identities of the species on the blot are indicated on the right side of the figure. Lanes: 1 – 16) His^+^ recombinants, P) His^-^ parent.

**C. Genomic Southern blots of independent His^-^ survivors.**

Genomic DNA from the parent strain and 16 independent His^-^ survivors was digested with *Bam*HI endonuclease, run on a 0.7% agarose gel, blotted to nylon, probed with a ^32^P-labeled 1.8 kb *HIS3* genomic clone and autoradiographed as described in the Materials and Methods. Locations of molecular weight markers are indicated on the left side of the figure and are marked in kilobase pairs. Identities of the species on the blot are indicated on the right side of the figure. Lanes: 1 – 16) His^-^ survivors, P) His^-^ parent.

**Figure S2. Wild-type Rad59, but not Rad59-Y92A or Rad59-K166A, interacts with Rad52.** Proteins were precipitated from yeast whole cell extracts with anti-FLAG antibody. Precipitated proteins where separated on SDS-PAGE gels, transferred to a PVDF membrane, and probed with anti-V5 antibody. Each blot displays the results from one of at least three independent experiments. (A) All strains expressed wild-type Rad59 with a C-terminal fusion of the V5 epitope as indicated on the left side of the blot. Above each lane is indicated whether each strain expressed untagged wild-type Rad52 or Rad59 proteins, FLAG-tagged wild-type Rad52, or FLAG-tagged wild-type or mutant Rad59. The lower blot displays the Rad59-V5 signals generated by probing 10% of the whole cell extracts prior to immunoprecipitation with anti-V5 antibody. (B) Same as in (A) except that each strain expressed the V5-tagged Rad59-Y92A mutant protein. (C) Same as in (A) except that each strain expressed the V5-tagged Rad59-K166A mutant protein.

**Figure S3. Levels of Rad52 are not reduced in *rad59* mutants.** Aliquots of whole cell extracts from wild-type and *rad59* mutant cells were subjected to SDS-PAGE, transferred to a PVDF membrane, and probed with anti-FLAG antibody. Each blot displays the results from one of at least three independent experiments.

**Figure S1**


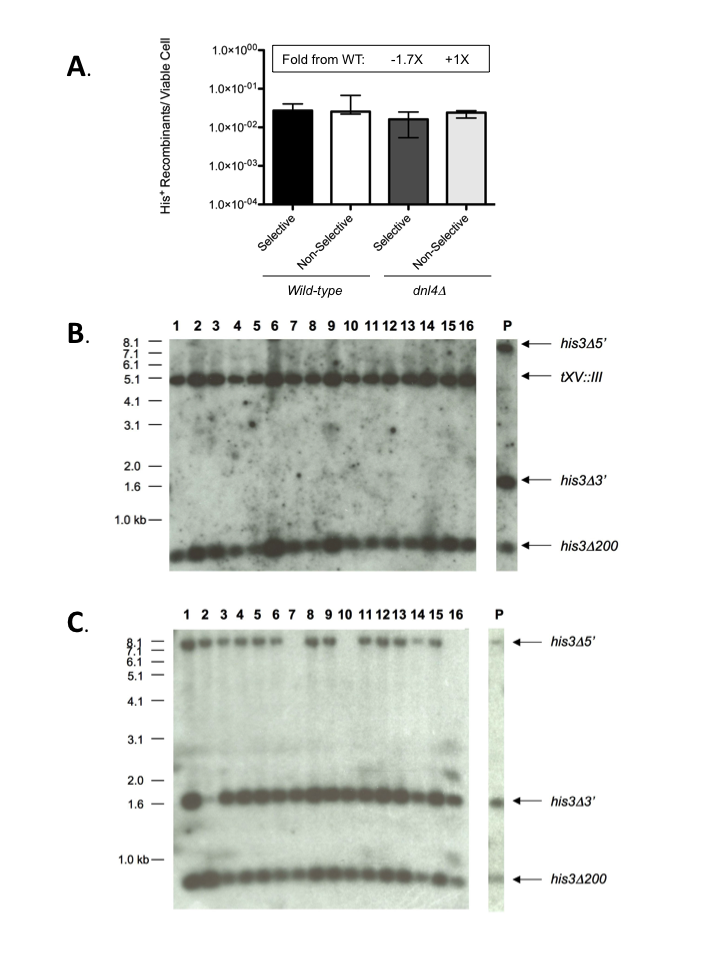


**Figure S2**


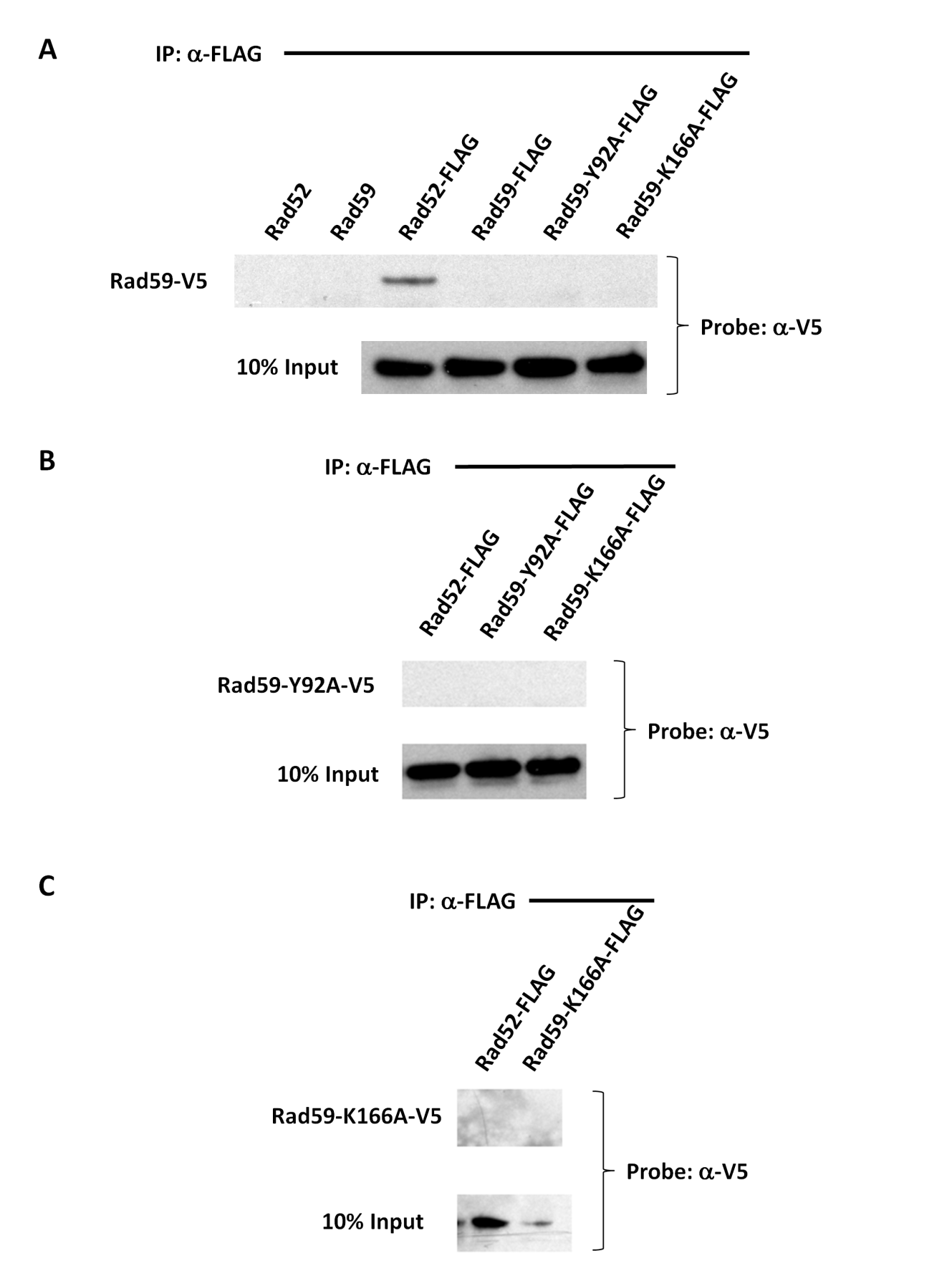


**Figure S3**

**
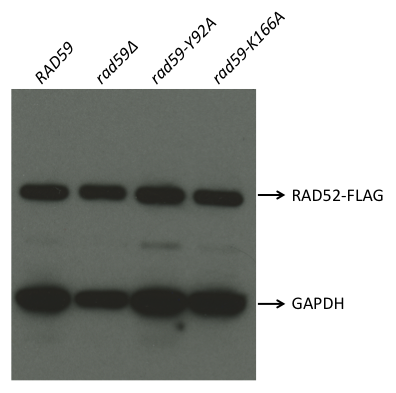
**

**Supplementary Table 1.** Plating efficiencies of wild-type and mutant diploid strains before and after HO-stimulated DSB formation on two chromosomes.

|  | **Plating efficiency (%) ^a^** | |
| --- | --- | --- |
| **Genotype** | **Pre-induction** | **Post-induction** |
| Wild-type | 49.2 (38.9, 87.8) | 34.6 (29.1, 50.4) |
| *rad51∆^-/-^* | 32.2 (29.8, 34.5) | 35.7 (17.9, 40.7) |
| *rad52∆^-/-^* | 12.2 (7.8, 23.7) | 9.6 (6.6, 16.2) |
| *rad59∆^-/-^* | 28.4 (25.1, 38.2) | 20.9 (11.4, 32.3) |
| *rad51∆^-/-^ rad59∆^-/-^* | 27.2 (21.7, 30.8) | 13.9 (7.4, 22.1) |
| *rad52∆^-/-^ rad59∆^-/-^* | 11.9 (10.0, 14.6) | 4.1 (1.9, 5.9) |

**^a^** Median plating efficiencies were determined before and after the induction of expression of HO-endonuclease from a minimum of 10 independent cultures as described in the Materials and Methods. The 95% confidence intervals are in parentheses.

**Supplemental Method**

**Plating efficiencies**

Plating efficiencies before and after DSB formation were assessed as described previously (Pannunzio et al., 2008). One ml cultures were inoculated with single colonies and grown overnight at 30°C. Cells were counted by hemacytometer, appropriate dilutions plated to YPD, and the number of colonies that arose after incubation at 30˚C for two to three days assessed. Dividing the number of colonies that arose by the number of cell bodies plated then multiplying by 100 was used to determine plating efficiency before DSB formation. Next, galactose was added to the cultures to a final concentration of 2%. After four more hours at 30°C to elicit DSB formation, cells were counted, appropriate dilutions plated onto YPD, and the number of colonies that arose after incubation at 30°C for two to three days counted. Plating efficiency following DSB formation was calculated as above. Median plating efficiencies and the 95% confidence intervals from a minimum of 10 independent trials were reported, and the Mann-Whitney test was used to assess statistical significance.
